# Towards Efficient Data Collection in Space-Based Internet of Things †

**DOI:** 10.3390/s19245523

**Published:** 2019-12-13

**Authors:** Changjiang Fei, Baokang Zhao, Wanrong Yu, Chunqing Wu

**Affiliations:** College of Computer, National University of Defense Technology, Changsha 410073, China; bkzhao@nudt.edu.cn (B.Z.); wlyu@nudt.edu.cn (W.Y.); wuchunqing@nudt.edu.cn (C.W.)

**Keywords:** sensory data collection, space-based Internet of Things, Internet of Things, spectral clustering, spatiotemporal compressive sensing

## Abstract

Due to the strong anti-destructive ability, global coverage, and independent infrastructure of the space-based Internet of Things (S-IoT), it is one of the most important ways to achieve a real interconnection of all things. In S-IoT, a single satellite can often achieve thousands of kilometers of coverage and needs to provide data transmission services for massive ground nodes. However, satellite bandwidth is usually low and the uplink and downlink bandwidth is extremely asymmetric. Therefore, exact data collection is not affordable for S-IoT. In this paper, an approximate data collection algorithm is proposed for S-IoT; that is, the sampling-reconstruction (SR) algorithm. Since the uplink bandwidth is very limited, the SR algorithm samples only the sensory data of some nodes and then reconstructs the unacquired data based on the spatiotemporal correlation between the sensory data. In order to obtain higher data collection precision under a certain data collection ratio, the SR algorithm optimizes the sampling node selection by leveraging the curvature characteristics of the sensory data in time and space dimensions. Moreover, the SR algorithm innovatively applies spatiotemporal compressive sensing (ST-CS) technology to accurately reconstruct unacquired sensory data by making full use of the spatiotemporal correlation between the sensory data. We used a real-weather data set to evaluate the performance of the SR algorithm and compared it with two existing representative approximate data collection algorithms. The experimental results show that the SR algorithm is well-suited for S-IoT and can achieve efficient data collection under the condition that the uplink bandwidth is extremely limited.

## 1. Introduction

The target of the Internet of Things (IoT) is to connect everything. The terrestrial IoT is mainly based on terrestrial networks, such as private networks, the Internet, and mobile communication networks. For this reason, because of the limitations of terrestrial network coverage, the scope of IoT applications is limited. For example, areas lacking terrestrial infrastructure, such as polar regions, oceans, and forests, make it difficult to deploy and apply large numbers of nodes. The advantages of space-based information networks include strong resistance to damage, global coverage, and infrastructure independence. Using a space-based information network as the network for IoT information transmission, building a space-based Internet of Things (S-IoT) is an effective way to realize the real interconnection of all things [1,2,3,4,5,6].

S-IoT is a comprehensive information system that is based on the space-based information network and provides interactions between things and things, people and things, and people and people. S-IoT is an extension and supplement to the terrestrial IoT. It mainly provides data transmission services for nodes in areas that are difficult to cover by terrestrial networks, such as forests, oceans, and deserts, as well as nodes in special areas, such as disaster areas and battlefields.

At present, the research on S-IoT has just started, and only a small amount of preliminary research has been carried out on data collection [7], application protocols [8], modulation schemes [9], and authentication protocols [10]. Despite this, S-IoT has attracted extensive attentions from many organizations including Inmarsat, Iridium, Globalstar and Orbcomm, and reports from Northern Sky Research (NSR) also show that in 2020, S-IoT’s revenue will likely be as high as $1.7 billion [11].

The foundation of S-IoT’s service for a variety of applications is data collection. Data collection is the primary operation in S-IoT. Data collection in S-IoT refers to the process of using the space-based information network to collect sensory data from ground nodes and store this in data centers.

However, there are huge challenges in S-IoT data collection. Firstly, in S-IoT, a single satellite usually covers a vast area and needs to provide data transmission services for massive ground nodes. The coverage of a terrestrial wireless base station is generally hundreds of meters to tens of kilometers. For example, in terrestrial IoT, such as long range radio (LoRa) or narrow band Internet of Things (NB-IoT), a base station can only cover tens of kilometers and provide services for hundreds of nodes. The coverage of a satellite (equivalent to a base station) is usually thousands of kilometers. For example, when the orbit altitude is 500 km and the half beam direction angle is $60^{\circ }$, the diameter of the satellite coverage area is about 2000 km. In such a wide coverage, the satellite usually needs to serve tens of thousands of nodes or even more. By 2025, the number of machine to machine (M2M) and IoT networks that are connected to the space-based information network are expected to reach 5.96 million [12].

Secondly, the bandwidth of the satellite–ground link is usually low, and the uplink and downlink bandwidth is extremely asymmetric. Due to the limitation of the power and weight of satellites, the bandwidth of the satellite–ground link is usually only on the order of Mbps and shared by massive nodes. Even worse, the uplink and downlink bandwidth of the satellite–ground link is significantly asymmetric because the transmission power and antenna size of the ground nodes are more limited compared to satellites. The uplink and downlink bandwidth ratio usually reaches 1:10 or even 1:100.

Therefore, the extremely limited uplink bandwidth of S-IoT will face competition by massive nodes, making it difficult to achieve complete and accurate data collection. This brings forth the important issues of approximate data collection in S-IoT. When we design approximate data collection algorithms for S-IoT, there are several important factors that need to be considered. Firstly, the sensory data in S-IoT are usually environmental parameters or location-related information, and there is a strong correlation between them in both time and space dimensions, so there is redundancy. Secondly, the ground nodes in S-IoT are usually distributed over a wide area, and there may not be stable links between nodes, or even no link at all. Therefore, nodes in S-IoT are often directly connected to satellites. Thirdly, the nodes in S-IoT are usually distributed in the wild and even carried by animals. Due to the limitations of weight, volume, and energy supply, the computing and storage resources of nodes are severely limited.

Therefore, an intuitive but very effective method of approximate data collection is to sample the sensory data of some nodes and then reconstruct the sensory data of the unsampled nodes by using the spatiotemporal correlation between the sensory data. For this method, in order to obtain higher data collection precision under a certain data collection ratio, we need to solve two problems: (1) how to optimize the sampling node selection during the sampling phase; (2) how to make full use of the spatiotemporal correlation between the sensory data during the reconstruction phase. Based on this idea, a general approximate data collection algorithm is proposed in this paper for S-IoT; that is, the sampling-reconstruction (SR) algorithm. The contributions of this paper can be summarized as follows:

- To the best of our knowledge, the proposed SR algorithm is the first approximate data collection algorithm for S-IoT, which is well suited for S-IoT and can achieve efficient data collection with severely limited uplink bandwidth.
- In order to obtain the highest possible data collection precision under a certain data collection ratio, we optimize the sampling node selection by using the curvature characteristics of the sensory data in time and space dimensions. Moreover, we innovatively use the spatiotemporal compressive sensing (ST-CS) technology to make full use of the spatiotemporal correlation of the sensory data to accurately reconstruct unacquired sensory data.
- The proposed SR algorithm is validated on a real-weather data set and compared with two existing representative approximate data collection algorithms in terrestrial IoT and wireless sensor network (WSN).

The following is an overview of the other chapters of this paper: In the Section 2, the work related to approximate data collection is reviewed. In the Section 3, the problem scenario of approximate data collection in S-IoT is described. In the Section 4, the SR algorithm that is proposed in this paper is elaborated, and the overall framework of the SR algorithm and the specific processes of clustering, sampling, and reconstruction are included. In the Section 5, the relevant performance of the SR algorithm is evaluated, and finally a summary of this paper and the future work are provided.

## 2. Related Work

As the research on S-IoT has just started, related research on approximate data collection in S-IoT has not been found. In [7], the problem of data collision and increased delay caused by a large number of ground nodes connected to a satellite in S-IoT is studied. However, the authors studied this from the perspective of access control rather than approximate data collection. In terrestrial IoT and WSN, many approximate data collection algorithms have been proposed [13,14]. According to [13], these algorithms consist of three major categories, namely model-based algorithms [15,16,17,18,19,20], compressed-sensing-based algorithms [21,22,23,24,25,26,27], and query-driven algorithms [28,29,30,31,32]. Below, we first discuss the differences between S-IoT and terrestrial IoT/WSN, then introduce the basic ideas and representative work of the three types of algorithms and analyze their advantages, disadvantages, and applicability in S-IoT.

### 2.1. S-IoT and Terrestrial IoT/WSN

Both S-IoT and terrestrial IoT/WSN acquire data through devices such as radio frequency identification (RFID) readers and sensors carried on the nodes, and then collect the data to support related applications. However, terrestrial IoT/WSN transmits data through terrestrial networks, such as the Internet, mobile communication networks, or private networks, while S-IoT transmits data through space-based information networks. Moreover, S-IoT is mainly used as a supplement to terrestrial IoT/WSN, and is used for environmental protection, animal monitoring, and other applications in areas where terrestrial networks are difficult to cover, such as in forests, oceans, and deserts; and for communication services in special areas, such as disaster areas and battlefields.

Due to the differences in transmission networks and application fields, S-IoT and terrestrial IoT/WSN have significant differences in aspects of base station coverage, internode links, and connection modes between nodes and base stations, as shown in Table 1.

As mentioned in Section 1, the coverage of a base station in terrestrial IoT/WSN is hundreds of meters to tens of kilometers, serving tens to hundreds of nodes. A satellite (equivalent to a base station) in S-IoT can usually cover a range of thousands of kilometers, and may need to serve tens of thousands of nodes or even more.

The nodes in terrestrial IoT/WSN usually form a network, such as the Internet of Vehicles or a wireless sensor network. The sensory data acquired by the node are transmitted to the base station through this network. Nodes and base stations are connected via multihop links between nodes. In the application fields of S-IoT, ground nodes are usually distributed over a wide area, and there may not be stable links between nodes, or even no link at all. For example, in animal monitoring, there may not be links between sensors carried by animals. Even if they exist, the links are difficult to stabilize due to animal migration. Therefore, nodes are usually directly connected to satellites.

The resources of the terrestrial IoT/WSN transmission network are relatively abundant, while the resources of the network formed by the ground nodes are severely limited due to the restrictions on the weight, volume, and energy supply of the nodes. Therefore, the main purpose of the approximate data collection algorithms in terrestrial IoT/WSN is to reduce the communication overhead and energy consumption of the internode network.

### 2.2. Model-Based Algorithms

In model-based algorithms, the correlation between sensory data is described by a mathematical model. Local prediction models are distributed and run on sensor nodes. In order to establish a global prediction model, the parameters of all local models are transmitted to the sink. If a sensory value and the predicted value of the local model are within a given error range, it does not need to be transmitted to the sink. The sink uses the value predicted by the global model as the collected value of the sensory data. Sensory data need to be transmitted to the sink only if the predicted values are not within the error range.

In order to minimize the communication from sensor nodes to the sink, the authors of [16] proposed Ken, an approximate technique based on replicated dynamic probabilistic models. A series of dynamic probability models are distributed in the sensor network. The sink synchronously runs all the same models. If sensor nodes find that newly arrived sensory values and the values predicted by the models are within the error range, these sensory data will not be sent to the sink. The sink computes the predictions of sensory data through the synchronized models and uses them as the approximation values. Otherwise, a subset of sensory data needs to be transmitted to the sink to update the models.

In [17], an adaptive sampling approach (ASAP) was developed. The sensor network is divided into multiple clusters by ASAP, and the nodes with close sensory values are assigned to one cluster. During data collection, for each cluster, some nodes are selected by ASAP as sampling nodes, and their values are collected directly. The values of the unsampled nodes in the cluster are predicted by the probability model. Probability models are constructed by exploiting the spatial and temporal correlations among sensory data. The method in [18] also divides the sensor network into clusters. The temporal correlations and spatial correlations of sensory data are described by linear regression models and correlation graphs, respectively. When collecting data, the sink will only receive data from the minimum subset of sensor nodes. The problem of determining the minimum subset of sensor nodes can be modeled as a minimum-dominating set problem.

Approximate data collection in body-area sensor networks was studied in [19]. Firstly, the proposed algorithm learns the temporal and spatial correlations among sensory data and a weighted directed graph is constructed to represent the transmission priorities of sensor nodes. Secondly, in the data collection process, the sensory values of the higher-priority sensor nodes are overheard by each node, and only the differences between the overheard values and its value are reported.

The model-based algorithms only need to transmit part of the sensory data by utilizing the temporal and spatial correlations among sensory data, thereby reducing communication overhead and energy consumption. However, firstly, since the model used by the algorithm is usually too ideal, the correlation between sensory data cannot be accurately described. Secondly, local models need to be run on sensor nodes, resulting in additional computation cost and energy consumption. Thirdly, for S-IoT, if the global model runs on the ground sink node, the communication overhead in the space-based information network will not decrease; if the global model runs in the data center of S-IoT, guaranteeing the consistency between local and global models will bring additional communication overhead to the network (including internode networks and space-based information networks).

### 2.3. Compressed-Sensing-Based Algorithms

Compressed-sensing-based algorithms assume that all nodes in the network report data synchronously, and the sensory data of all nodes over a period of time can constitute a m×n matrix, where m refers to the number of data collections in the time period and n refers to the number of nodes in the network. The algorithms assume that the sensory data matrix is sparse in a certain subspace. The algorithms first map the sensory data to the above-mentioned sparse subspace to compress the data. Subsequently, the compressed data is sent to the sink, which then recovers the original data through the compressed data according to the sparse characteristics of sensory data.

The work in [23] proposed the compressive data gathering (CDG) algorithm, which is the first complete design to apply a compressive sampling theory to sensory data collection for large-scale wireless sensor networks. In [23], each sensor node generates a data vector by multiplying its sensory value and a random vector. In the routing tree, each node transmits the sum of its data vector and data vectors of all its child nodes. Based on the theory of compressive sampling, the sink can recover the original sensory data through the received data vectors and the random matrix that consists of all the random vectors.

Authors of [22] found a matrix that has a good restricted isometry property and used this matrix to compress sensory data during collection. Furthermore, in order to fully utilize the sparsity of sensory data, different sparsity patterns can be utilized by the proposed algorithm.

The algorithm proposed in [26] firstly gathers a small number of compressive sensing measurements through random walk routing in order to reduce energy consumption. Then, all original sensory data are recovered according to these measurements.

Conventional clustered compression algorithms usually ignore data correlation among different clusters. The algorithm designed in [27] provides better data collection performance through exploring spatial correlations among different clusters and developing a detailed solution method to recover the original data.

When the sensory data matrix has a high sparsity in a certain subspace, the compressed-sensing-based algorithms can effectively compress the sensory data, thereby reducing communication overhead and energy consumption. However, firstly, the assumption that the sensory data matrix is sparse in a certain subspace is too strong. Even if the above assumption is correct, it is difficult to find the subspace in which the sensory data is sparse. Secondly, data compression operations need to be performed on the sensor nodes, resulting in additional computational overhead. Thirdly, for S-IoT, if the data is transmitted directly to the satellite after being compressed at sensor nodes, the amount of data that the space-based information network needs to transmit may increase; if the compressed sensory data are first transmitted to the ground sink node and then transmitted to the satellite, it needs to rely on the internode network.

### 2.4. Query-Driven Algorithms

In query-driven algorithms, the network sends the required data to the sink according to the input query and precision requirements. Since the algorithm is designed for a specific query, usually only a small portion of sensory data needs to be transmitted, thus the amount of data transmitted is greatly reduced.

Top-k queries are popular statistical queries [28] proposed to use historical sensory data to optimize top-k queries and formulate the optimization problem as a linear program. In general, if the historical sensory value of a node is larger, the probability of it being sampled is also greater.

Sensory data aggregation helps users understand the overall situation in a given area. One study [29] proposed a Bernoulli-sampling-based approximate aggregation algorithm, which can satisfy the arbitrary precision requirement. In order to track quantiles and range counts in wireless sensor networks, [32] proposed a dynamic binary-tree-based deterministic tracking algorithm to track approximate quantiles (ε,ϕ) and a Bernoulli-sampling-based algorithm to track approximate range counts (ε,δ).

The query-driven algorithms take into account the needs of users and only need to transmit a small amount of necessary data, thereby greatly reducing communication overhead and energy consumption. However, such algorithms are only designed for a particular type of query and are not general data collection algorithms.

### 2.5. Summary

In the previous subsections, we analyzed the advantages and disadvantages of each type of algorithm and the problems that existed in applying them to S-IoT. Since the query-driven algorithms are not general approximation data collection algorithms, we do not focus on them. In general, model-based algorithms and compressed-sensing-based algorithms mainly have two problems when applied to S-IoT: (1) the main purpose of these two type of algorithms is to reduce the communication overhead and energy consumption of the internode network, and the implementation of the algorithms usually also depends on the internode network; (2) these two type of algorithms will bring additional overhead to the ground nodes. Therefore, the existing approximate data collection algorithms in terrestrial IoT/WSN are not suitable for S-IoT.

## 3. Problem Scenario

**Figure 1:**
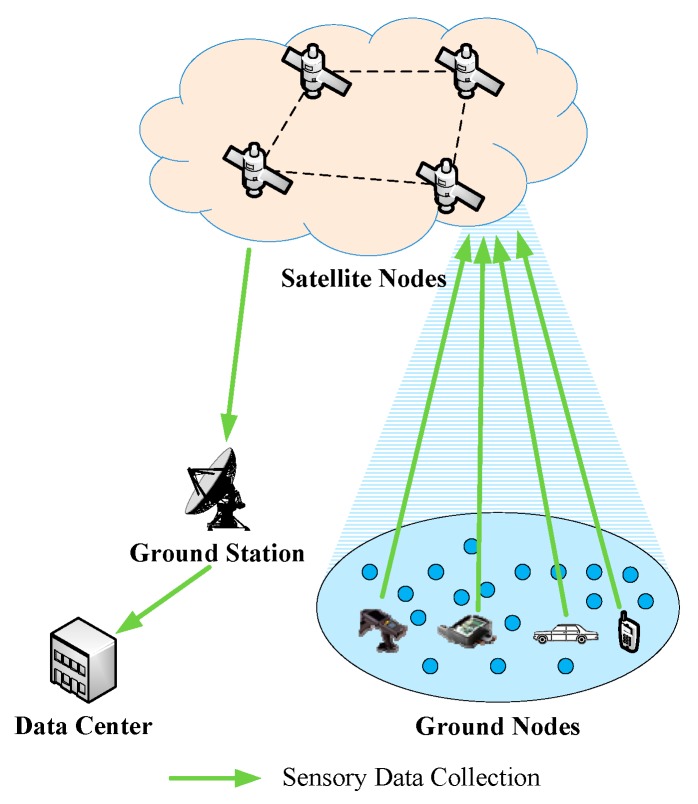
The data collection of S-IoT.

As shown in Figure 1, when S-IoT collects sensory data, the satellite node first receives the sensory data from ground nodes, and then the sensory data are sent to the data center through the ground station.

**Figure 2:**
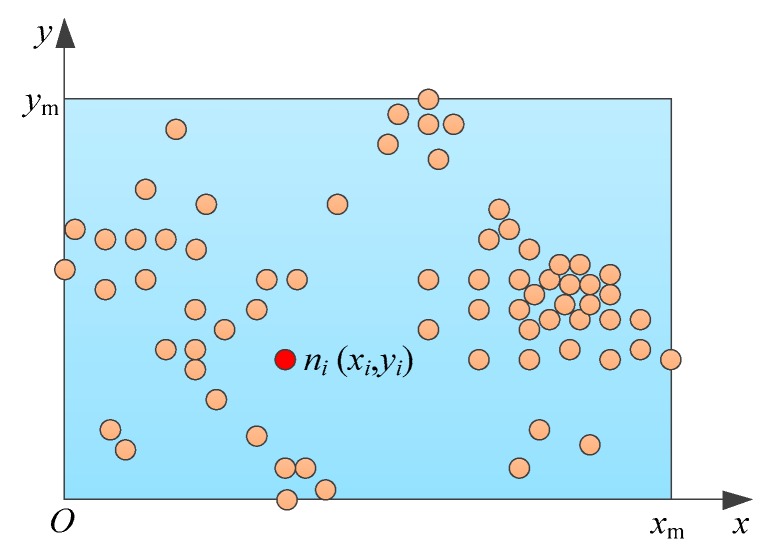
Ground nodes in a data collection task.

In a data collection task, there are many ground nodes in a specific geographic area. As shown in Figure 2, the rectangle refers to the specific distribution area of the ground nodes in the data collection task, and N is the number of nodes. A two-dimensional coordinate system xOy is established, and the lower left corner of the rectangular area is taken as the origin. In the rectangular area, $x_{m}$ is the maximum value of the horizontal coordinate and $y_{m}$ is the maximum value of the vertical coordinate. We can obtain the coordinate of each node through the positioning device on the node. At the moment t, we can use $\left(x_{i}(t),y_{i}(t)\right)$ ($x_{i}(t)\in [0,x_{m}]$, $y_{i}(t)\in [0,y_{m}]$) to represent the coordinate of node $n_{i}$(i=1,⋯,N).

We use $t_{0}$ to indicate a certain data collection moment. At the moment $t_{0}$, the sensory data of node $n_{i}$ can be expressed as $d_{i}(t_{0})$ and sensory data in different data collection cycles can be represented as a series

(1) $$\cdots ,d_{i}(t_{0}-T),d_{i}(t_{0}),d_{i}(t_{0}+T),\cdots ,$$

where T refers to the data collection period. Here, T is determined by specific data collection tasks. At time $t_{0}$, $\left\{d_{1}(t_{0}),\cdots ,d_{N}(t_{0})\right\}$ is the collection of sensory data for all nodes.

In each data collection cycle, only the sensory data of some nodes will be collected by the SR algorithm, and the unobtained sensory data are reconstructed. When the data collection moment is $t_{0}$, we use M (M≤N) to represent the number of nodes collecting sensory data, and $\left\{d_{1}^{\prime }(t_{0}),\cdots ,d_{N}^{\prime }(t_{0})\right\}$ refers to the reconstructed sensory data. Therefore, r=M/N means the data collection ratio. Data collection precision is expressed as

(2) $$A=1-\frac{1}{N}\sum_{n=1}^{N}\frac{\left|d_{n}^{\prime }(t_{0})-d_{n}(t_{0})\right|}{\left|d_{n}(t_{0})\right|+\alpha }$$

In the above formula, in order to avoid a calculation error when $\left|d_{n}(t_{0})\right|=0$, α is set to a small positive number. The reconstruction method, sampling method, and data collection ratio can affect data collection precision. The SR algorithm needs to improve the data collection precision as much as possible under a certain data collection ratio.

## 4. Sampling-Reconstruction Algorithm

In this section, the overall framework of the SR algorithm is presented first, and then the basic processes of clustering, sampling, and reconstruction in the SR algorithm are elaborated.

### 4.1. Overview

The basic idea of the SR algorithm is to collect only part of the sensory data and reconstruct the uncollected data in the data center by leveraging the spatiotemporal correlations of sensory data. Therefore, intuitively, the SR algorithm should include two main processes of sampling and reconstruction. However, in S-IoT, a data collection task may contain a large number of nodes. If all nodes participate in the operation during the sampling and reconstruction phases, the computational complexity will be greatly increased. In addition, only the nodes in the neighboring area have strong spatial correlation. Therefore, before performing the sampling and reconstruction operations, we first cluster the nodes into a series of clusters with strong spatial correlation.

**Figure 3:**
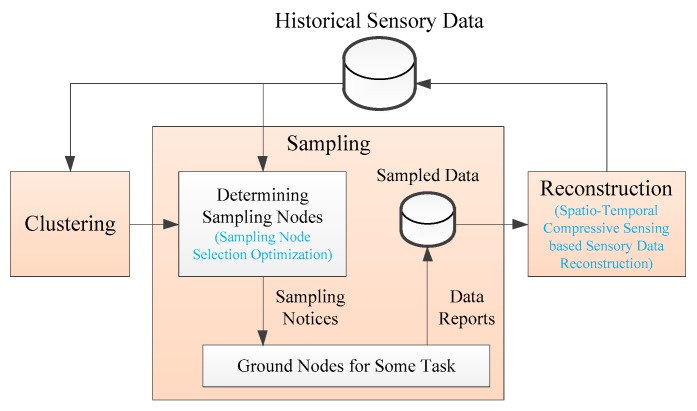
The framework of the sampling-reconstruction (SR) algorithm.

Figure 3 is the framework of the SR algorithm. The SR algorithm is mainly composed of three phases: clustering, sampling, and reconstruction. For the clustering phase, ground nodes are clustered into many clusters that have strong spatial correlation according to historical sensory data that are stored in the historical sensory data repository. Each cluster needs to perform the subsequent reconstruction process separately.

For the sampling phase, the nodes that need to report the sensory data are first determined, then the sampled nodes receive the sampling notices, and eventually the data center receives the sensory data reported from the sampled nodes. The advantages of satellite broadcasting can be fully utilized in the transmission of sampling notices. In the determination of sampling nodes, in order to obtain higher data collection precision under a certain data collection ratio, we optimizes sampling node selection use the curvature characteristics of historical sensory data in time and space dimensions.

For the reconstruction phase, the spatiotemporal correlation between the sensory data is utilized by the SR algorithm, and the sensory data reconstruction of the unacquired nodes is implemented according to the acquired sensory data. In order to make full use of the spatiotemporal correlation of the sensory data to obtain higher data reconstruction precision, we innovatively adopt ST-CS technology in the reconstruction phase. The historical sensory data repository stores reconstructed sensory data for subsequent data collection and application services.

### 4.2. Clustering

For the time dimension, there is a strong correlation between the sensory data of a node in a neighboring time period $T_{C}$. The value of $T_{C}$ is related to the type of sensory data. For the spatial dimension, it can be intuitively found that compared with the distant nodes, the correlation between the sensory data of neighboring nodes is stronger. Nevertheless, it is not accurate to only rely on the distance between the nodes to determine the correlation of sensory data. For instance, suppose there are two adjacent nodes, one in the lawn and the other one located in the woods. The light intensity between the two nodes will be significantly different. Therefore, for the node clustering, we consider not only the positions of nodes but also the sensory values of nodes.

We cluster the nodes based on the reconstructed sensory data in the previous period, so that the nodes can be divided into a series of clusters, which have a strong spatial correlation. The spectral clustering algorithm is adopted. For a particular data collection moment $t_{0}$, $P=\{p_{1},\cdots ,p_{N}\}$ is the clustering sample set, where $p_{i}=\left(x_{i}(t_{0}-T),y_{i}(t_{0}-T),d_{i}^{\prime }(t_{0}-T)\right)$ (i=1,⋯,N) is the sample point. Algorithm 1 shows the specific process of node clustering. In this algorithm, $K^{\prime }$ refers to the dimension after dimensionality reduction, K refers to the dimension after clustering, and σ refers to Gaussian kernel function; $C_{1},\cdots ,C_{K}$ refer to the clusters after clustering, while $C_{k}$(k=1,⋯,K) is the subset of P, $C_{k_{i}}\cap C_{k_{j}}=\emptyset$ ($k_{i},k_{j}=1,\cdots ,K$, and $k_{i}\neq k_{j}$), and $C_{1}\cup C_{2}\cup \cdots \cup C_{K}=P$. The Ncut method is used by the algorithm.

Assuming the number of nodes in cluster $C_{k}$ is $N_{k}$, then the sensory data of all nodes in $C_{k}$ at $t_{0}$ and previous $T_{C}$ can be expressed as a $L\times N_{k}$ matrix

(3) $$D_{k}(t_{0})=\left[\begin{array}{cccc} d_{k_{1}}(t_{0}) & d_{k_{2}}(t_{0}) & \cdots & d_{k_{N_{k}}}(t_{0})\\ d_{k_{1}}(t_{0}-T) & d_{k_{2}}(t_{0}-T) & \cdots & d_{k_{N_{k}}}(t_{0}-T)\\ \vdots & \vdots & \ddots & \vdots \\ d_{k_{1}}(t_{0}-(L-1)T) & d_{k_{2}}(t_{0}-(L-1)T) & \cdots & d_{k_{N_{k}}}(t_{0}-(L-1)T) \end{array}\right],$$

where $L=\left\lfloor T_{C}/T\right\rfloor +1$ refers to the number of data collection, while the serial numbers of the nodes in $C_{k}$ are represented by $k_{1},\cdots ,k_{N_{k}}$. Here, $D_{k}(t_{0})$ is the $C_{k}$‘s sensory data matrix at $t_{0}$.
**Algorithm 1** Node Clustering**Input:**P, $K^{\prime }$, K, N, σ **Output:**$C_{1},\cdots ,C_{K}$/* Constructs adjacency matrixW */**for each**i∈{1,⋯,N}**do****for each**j∈{1,⋯,N}**do**$w_{ij}=\exp\left(-{\left\|p_{i}-p_{j}\right\|}_{2}^{2}/2\sigma^{2}\right)$**end for****end for**$W={\left(w_{ij}\right)}_{N\times N}$/* Constructs degree matrix**d** */**for each**i∈{1,⋯,N}**do**$d_{i}=\sum_{j=1}^{N}w_{ij}$**end for**$D=\mathrm{diag}(d_{1},d_{2},\cdots ,d_{N})$L=D−W$\left\{f_{1},\cdots ,f_{K^{\prime }}\}=Eigenvector(D^{-1/2}LD^{-1/2},K^{\prime }\right)$$F=(f_{1},\cdots ,f_{K^{\prime }})$/* StandardizesF*by row and generates*$F^{*}$ */**for each**i∈{1,⋯,N}**do****for each**$j\in \{1,\cdots ,K^{\prime }\}$**do**$f_{ij}^{*}=f_{ij}/\sqrt{\sum_{k^{\prime }=1}^{K^{\prime }}f_{ik^{\prime }}^{2}}$**end for****end for**$F^{*}={\left(f_{ij}^{*}\right)}_{N\times K^{\prime }}$/* *Constructs new samples after reducing dimensionality* */**for each**i∈{1,⋯,N}**do**${p^{\prime }}_{i}={\left(f_{i1}^{*},\cdots ,f_{iK^{\prime }}^{*}\right)}^{T}$**end for**$P^{\prime }=\{{p^{\prime }}_{1},\cdots ,{p^{\prime }}_{N}\}$$\left\{C_{1},\cdots ,C_{K}\}=Kmeans(P^{\prime },K\right)$**return**$C_{1},\cdots ,C_{K}$

### 4.3. Sampling

In order to obtain higher data collection precision under a certain data collection ratio, we will optimize the selection of sampling nodes.

It can be intuitively found that if the sensory data of a certain node changes greatly in the most recent period, or if there are significant differences between the sensory data of a certain node and the sensory data of its neighboring nodes, the more so the sensory data needs to be reported and the sampling priority should be higher. Since the sensory data of these nodes differs greatly from their historical values or the sensory data of nearby nodes, the difficulty of reconstruction is relatively high. The above two features can be characterized by the curvature characteristics of the sensory data in time and space dimensions. The larger the curvatures of time and space dimensions, the higher the sampling priority of the node. If the number of sampled nodes is M, we select the M nodes with the largest composite curvature of the time dimension and the spatial dimension as the sampled nodes.

Next, the curvature of the sensory data in the time dimension and the space dimension are calculated separately, and finally the composite curvature is obtained.

#### 4.3.1. Time Dimension Curvature

For the time dimension, an interpolation curve is firstly obtained based on the sensory data reconstructed at $t_{0}-T$ and previous $T_{C}$. We use the mean value of curvatures of the interpolation curve at each interpolation point (except for the two endpoints) as the time dimension curvature. Table 2 shows the reconstructed sensory data of $n_{i}(i=1,\cdots ,N)$ at $t_{0}-T$ and the previous $T_{C}$.

Based on the data in Table 2, the interpolation is performed in $\left[t_{0}-(L-1)T,t_{0}-T\right]$. Cubic spline interpolation is used as the interpolation method, which adopts natural boundary conditions. The following is the segmentation expression for the cubic spline interpolation function:

(4) $$\begin{array}{ll} s(t) & =d_{i}^{\prime }(t_{0}-lT)+\left\{d_{i}^{\prime }[t_{0}-lT,t_{0}-(l-1)T]-\left(\frac{1}{3}M_{l}+\frac{1}{6}M_{l-1}\right)h_{l}\right\}(t-t_{0}+lT)\\ & +\frac{1}{2}M_{l}{\left(t-t_{0}+lT\right)}^{2}+\frac{1}{6h_{l}}(M_{l-1}-M_{l}){\left(t-t_{0}+lT\right)}^{3}\\ & \left(t\in [t_{0}-lT,t_{0}-(l-1)T],l=L-1,\cdots ,2\right) \end{array}$$

in which

(5) $$d_{i}^{\prime }[t_{0}-lT,t_{0}-(l-1)T]=\frac{d_{i}^{\prime }(t_{0}-lT)-d_{i}^{\prime }(t_{0}-(l-1)T)}{\left(t_{0}-lT)-(t_{0}-(l-1)T\right)}=\frac{d_{i}^{\prime }(t_{0}-(l-1)T)-d_{i}^{\prime }(t_{0}-lT)}{T},\;h_{l}=(t_{0}-(l-1)T)-(t_{0}-lT)=T,\;M_{l}=s^{\prime \prime }(t_{0}-lT)\;(l=L-1,\cdots ,1).$$

According to natural boundary conditions, $M_{L-1}=0$, $M_{1}=0$. $M_{L-2},\cdots ,M_{2}$ can be obtained through the following formula:

(6) $$\left[\begin{array}{ccccc} 2 & \lambda_{L-2} & & & \\ \mu_{L-3} & 2 & \lambda_{L-3} & & \\ & \ddots & \ddots & \ddots & \\ & & \mu_{3} & 2 & \lambda_{3}\\ & & & \mu_{2} & 2 \end{array}\right]\left[\begin{array}{c} M_{L-2}\\ M_{L-3}\\ \vdots \\ M_{3}\\ M_{2} \end{array}\right]=\left[\begin{array}{c} d_{L-2}\\ d_{L-3}\\ \vdots \\ d_{3}\\ d_{2} \end{array}\right],$$

in which

(7) $$\lambda_{l}=1-\mu_{l},\;\mu_{l}=\frac{h_{l+1}}{h_{l+1}+h_{l}}=\frac{1}{2},\;\begin{array}{l} d_{l}=6d_{i}^{\prime }[t_{0}-(l+1)T,t_{0}-lT,t_{0}-(l-1)T]\\ \;=6\frac{d_{i}^{\prime }[t_{0}-(l+1)T,t_{0}-lT]-{d^{\prime }}_{i}[t_{0}-lT,t_{0}-(l-1)T]}{\left(t_{0}-(l+1)T)-(t_{0}-(l-1)T\right)}\\ =3\frac{{d^{\prime }}_{i}[t_{0}-lT,t_{0}-(l-1)T]-{d^{\prime }}_{i}[t_{0}-(l+1)T,t_{0}-lT]}{T} \end{array}$$

The curvature of interpolation curve s(t) at $t_{0}-lT$(l=L−2,⋯,2) is

(8) $$c_{i}(t_{0}-lT)=\frac{\left|M_{l}\right|}{{\left(1+{\left(s^{\prime }(t_{0}-lT)\right)}^{2}\right)}^{\frac{3}{2}}},$$

in which

(9) $$s^{\prime }(t_{0}-lT)=d_{i}^{\prime }[t_{0}-lT,t_{0}-(l-1)T]-\left(\frac{1}{3}M_{l}+\frac{1}{6}M_{l-1}\right)h_{l}$$

Therefore, the mean value of curvatures of s(t) at $t_{0}-(L-2)T,\cdots ,t_{0}-2T$ is

(10) $${\bar{c}}_{i}=\frac{1}{L-3}\sum_{l=2}^{L-2}c_{i}(t_{0}-lT)$$

#### 4.3.2. Spatial Dimension Curvature

The spatial dimension curvature is calculated separately in each cluster. The Gaussian curvature of the interpolation surface at each node at $t_{0}-T$ is used as the spatial dimension curvature. If the number of nodes is larger, the overhead of two-dimensional interpolation will be greater. Furthermore, we only need to obtain the curvature characteristics. Therefore, the curvature is estimated directly, and it is very similar with curvature estimation for a point-based surface. A simple estimation method proposed in [33] is used, also known as the Voronoi element method. We use $P_{k}=\{p_{k_{1}},\cdots ,p_{k_{N_{k}}}\}$ to represent the data points in $C_{k}$, and $p_{i}=\left(x_{i}(t_{0}-T),y_{i}(t_{0}-T),d_{i}^{\prime }(t_{0}-T)\right)$ ($i=k_{1},\cdots ,k_{N_{k}}$) is a data point.

For data point $p_{i}$, the following is the detailed process for its curvature estimation, which is performed by the Voronoi element method.

Estimating the normal vector: Firstly, a possible neighbor set ${\tilde{N}}_{i}=\{p_{j}|\left\|p_{i}-p_{j}\right\|<r_{i}\}$ of $p_{i}$ is collected with a distance threshold $r_{i}>0$. If $p_{i_{1}},\cdots ,p_{i_{m}}$ is the elements of ${\tilde{N}}_{i}$ ($p_{i}$ is not included), we can use the following formula to express the covariance matrix of the neighbors of $p_{i}$:

(11) $$C={\left[\begin{array}{c} p_{i_{1}}-{\bar{p}}_{i}\\ \cdots \\ p_{i_{m}}-{\bar{p}}_{i} \end{array}\right]}^{T}\left[\begin{array}{c} p_{i_{1}}-{\bar{p}}_{i}\\ \cdots \\ p_{i_{m}}-{\bar{p}}_{i} \end{array}\right].$$

In the above formula, ${\bar{p}}_{i}$ refers to the center of $p_{i_{1}},\cdots ,p_{i_{m}}$. A valid estimation of the normal vector of $p_{i}$ is the eigenvector $v_{\min}$ corresponding to the minimum eigenvalue $\lambda_{\min}$ of C.

Determining the set of neighbors: We project the points in ${\tilde{N}}_{i}$ to the tangent plane of $p_{i}$ and produce $P(p_{i})$. Then, we perform Delaunay triangulation for $P(p_{i})$ and get a graph $T_{i}$. The neighbor set of $p_{i}$ is $N_{i}=\{p_{j}|p_{j}\;\mathrm{is}\;\mathrm{the}\;\mathrm{neighbor}\;\mathrm{of}\;p_{i}\;\mathrm{in}\;T_{i}\}$.
**Algorithm 2** Sampling Node Selection**Input:**N, M, $T_{i}$(i=1,⋯,N), $D_{i}$(i=1,⋯,N), $t_{0}$, L, T, K, $\left\{k_{1},\cdots ,k_{N_{k}}\}(k=1,\cdots ,K\right)$, $r_{i}$(i=1,⋯,N), $w_{1}$, $w_{2}$ **Output:**$s_{1},\cdots ,s_{M}$/* Calculating time dimension curvature${\bar{c}}_{i}$ */**for each**i∈{1,⋯,N}**do**$s(t)=CubicSpline\left(T_{i},D_{i}\right)$ ($t\in [t_{0}-(L-1)T,t_{0}-T]$)**for each**l∈{L−2,⋯,2}**do**$M_{l}=s^{\prime \prime }(t_{0}-lT)$$c_{i}(t_{0}-lT)=\left|M_{l}\right|/{\left(1+{\left(s^{\prime }(t_{0}-lT)\right)}^{2}\right)}^{3/2}$**end for**${\bar{c}}_{i}=\left(\sum_{l=2}^{L-2}c_{i}(t_{0}-lT)\right)/(L-3)$**end for**/* Calculatingspatialdimensioncurvature$k_{G}(p_{i})$ */**for each**k∈{1,⋯,K}**do****for each**$i\in \{k_{1},\cdots ,k_{N_{k}}\}$**do**/* Estimating the normal vector$v_{\min}$ */${\tilde{N}}_{i}=\{p_{i_{1}},\cdots ,p_{i_{m}},p_{i}\}=\{p_{j}|\left\|p_{i}-p_{j}\right\|<r_{i}\}$${\bar{p}}_{i}=(p_{i_{1}}+\cdots +p_{i_{m}})/m$$C={\left(p_{i_{1}}-{\bar{p}}_{i}\right)}^{2}+\cdots +{\left(p_{i_{m}}-{\bar{p}}_{i}\right)}^{2}$$v_{\min}=MinEigenvector(C)$/* Determining the set of neighbors$N_{i}$ */$P(p_{i})=Project({\tilde{N}}_{i},p_{i})$$T_{i}=Delaunay(P(p_{i}))$$N_{i}=\{p_{j}|p_{j}\;\mathrm{is}\;\mathrm{the}\;\mathrm{neighbor}\;\mathrm{of}\;p_{i}\;\mathrm{in}\;T_{i}\}$/* *Calculating Gaussian curvature* */$T_{i}^{\prime }=TriGrid(p_{i},N_{i})$$\theta =SumAngle({T^{\prime }}_{i})$$A_{\mathrm{mixed}}=AreaMixed(p_{i})$$k_{G}(p_{i})=(2\pi -\theta )/A_{\mathrm{mixed}}$**end for****end for****for each**i∈{1,⋯,N}**do**$k_{C_{i}}=w_{1}{\bar{c}}_{i}+w_{2}k_{G}(p_{i})$**end for**$\left\{s_{1},\cdots ,s_{M}\}=SelectMax(k_{C_{1}},\cdots ,k_{C_{N}},M\right)$**return**$s_{1},\cdots ,s_{M}$

**Figure 4:**
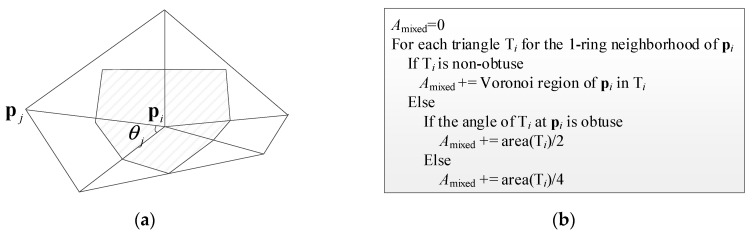
Gaussian curvature calculation. (**a**) A triangular grid diagram composed of $p_{i}$ and its neighbors; (**b**) the calculation method of $A_{\mathrm{mixed}}$.

Calculating the Gaussian curvature: Figure 4a is a triangular grid diagram composed of $p_{i}$ and its neighbors. According to [34], the Gaussian curvature at $p_{i}$ can be estimated by

(12) $$k_{G}(p_{i})=\frac{2\pi -\sum_{j=1}^{\#f}\theta_{j}}{A_{\mathrm{mixed}}},$$

in which #f is the number of triangles including $p_{i}$, $\theta_{j}$ is shown in Figure 4a, $A_{\mathrm{mixed}}$ is the area of the shaded portion in Figure 4a, and can be calculated according to the method in Figure 4b.

Therefore, the composite curvature of node $n_{i}(i=1,\cdots ,N)$ in the time dimension and the spatial dimension is

(13) $$k_{C_{i}}=w_{1}{\bar{c}}_{i}+w_{2}k_{G}(p_{i}),$$

in which $w_{1}\geq 0$ and $w_{2}\geq 0$ are the weights of time dimension curvature and spatial dimension curvature in the composite curvature, respectively.

The sampling node selection process is shown in Algorithm 2. In this algorithm, $T_{i}=\left(t_{0}-(L-1)T,t_{0}-(L-2)T,\cdots ,t_{0}-T\right)$, $D_{i}=\left(d_{i}^{\prime }(t_{0}-(L-1)T),d_{i}^{\prime }(t_{0}-(L-2)T),\cdots ,d_{i}^{\prime }(t_{0}-T)\right)$, and $s_{1},\cdots ,s_{M}$ are the serial numbers of M sampling nodes. Here, CubicSpline(⋅) is a cubic spline interpolation function, MinEigenvector(⋅) is a function of finding the eigenvector corresponding to the minimum eigenvalue of a matrix, $Project({\tilde{N}}_{i},p_{i})$ projects the points in set ${\tilde{N}}_{i}$ to the tangent plane of point $p_{i}$, Delaunay(⋅) is a Delaunay triangulation function, TriGrid(⋅) calculates a triangular grid diagram composed by a point and its neighbors, $SumAngle({T^{\prime }}_{i})$ calculates the sum of the angles of the triangles containing $p_{i}$ in ${T^{\prime }}_{i}$ at $p_{i}$, AreaMixed(⋅) is a function that calculates $A_{\mathrm{mixed}}$ for a point, and $SelectMax(k_{C_{1}},\cdots ,k_{C_{N}},M)$ finds the serial numbers of the M nodes with the largest composite curvature.

### 4.4. Reconstruction

Data collected from real-world applications usually exhibits a certain structure or redundancy, for example, the values of adjacent rows or columns in the sensory data matrix are relatively close. With this feature, the ST-CS can accurately reconstruct missing values in the data set from partial observations. ST-CS has been applied in the reconstruction of missing values for Internet traffic measurements [35], wireless sensor networks [36], and trajectory data [37]. The principle of ST-CS is described in detail in [35].

If obvious low-rank structure (that is, redundancy) and spatiotemporal stability [35,36] can be revealed in the sensory data, the sensory data can be effectively reconstructed by ST-CS technology. The sensory data in S-IoT are usually environmental parameters or location-related information that satisfy the above characteristics.

For sensory data matrix $D_{k}(t_{0})$ of $C_{k}$ at $t_{0}$, we define a $L\times N_{k}$ sampling indicator matrix

(14) $$S_{k}(t_{0})={\left(s_{i}(t)\right)}_{L\times N_{k}}=\left\{ \begin{array}{l} 1\;\mathrm{if}\;d_{i}(t)\;\mathrm{in}\;D_{k}(t_{0})\;\mathrm{been}\;\mathrm{sampled}\\ 0\;\mathrm{otherwise} \end{array}\right.,$$

which implies whether sensory data in $D_{k}(t_{0})$ has been sampled.

Assuming the reconstructed sensory data matrix is ${\hat{D}}_{k}(t_{0})$, then ${\hat{D}}_{k}(t_{0})$ can be expressed as the following form through singular value decomposition:

(15) $${\hat{D}}_{k}(t_{0})=LR^{*}.$$

According to [35], the following optimization problem can be used to express the sensory data reconstruction problem:

(16) $$\min\left\{{\left\|S_{k}(t_{0})\cdot (LR^{*})-D_{k}(t_{0})\right\|}_{F}^{2}+\lambda \left({\left\|L\right\|}_{F}^{2}+{\left\|R^{*}\right\|}_{F}^{2}\right)+{\left\|H(t_{0})LR^{*}\right\|}_{F}^{2}+{\left\|LR^{*}T\right\|}_{F}^{2}\right\}$$

In the above formula, λ refers to the Lagrangian multiplier, and ${\left\|\cdot \right\|}_{F}^{2}$ refers to the Frobenius (Euclidean) paradigm. $H(t_{0})$ refers to the space constraint matrix, while T refers to the time constraint matrix, which will be described in detail below. Through adjusting λ, with this optimization problem, we can estimate L and $R^{*}$; after this, ${\hat{D}}_{k}(t_{0})$ is obtained.
**Algorithm 3** Sensory Data Reconstruction**Input:**K, $D_{k}(t_{0})$(k=1,⋯,K), L, $N_{k}$, $\left\{k_{1},\cdots ,k_{N_{k}}\right\}$(k=1,⋯,K), $t_{0}$, T, T **Output:**${\hat{D}}_{1}(t_{0}),\cdots ,{\hat{D}}_{K}(t_{0})$**for each**k∈{1,⋯,K}**do**/* Constructssampling indicator matrix$S_{k}(t_{0})$ */**for each**l∈{0,⋯,L−1}**do****for each**$i\in \{k_{1},\cdots ,k_{N_{k}}\}$**do****if**$d_{i}(t_{0}-lT)$ in $D_{k}(t_{0})$ been sampled$s_{i}(t_{0}-lT)=1$**else**$s_{i}(t_{0}-lT)=0$**end if****end for****end for**$S_{k}(t_{0})={\left(s_{i}(t_{0}-lT)\right)}_{L\times N_{k}}$/* Derive space constraint matrix$H(t_{0})$ */**for each**i∈{1,⋯,N}**do****for each**j∈{1,⋯,N}**do****if**$n_{i}$ and $n_{j}$ are neighbors at $t_{0}$${h^{\prime }}_{i,j}(t_{0})=1$**else**${h^{\prime }}_{i,j}(t_{0})=0$**end if****end for****end for**$H^{\prime }(t_{0})={\left({h^{\prime }}_{i,j}(t_{0})\right)}_{N\times N}$**for each**i∈{1,⋯,N}**do**$temp=\sum_{j=1}^{N}{h^{\prime }}_{i,j}(t_{0})$**for each**j∈{1,⋯,N}**do****if**temp=0$h_{i,j}(t_{0})=0$**else if**i=j$h_{i,j}(t_{0})=1$**else**$h_{i,j}(t_{0})=-{h^{\prime }}_{i,j}(t_{0})/temp$**end for****end for**$H(t_{0})={\left(h_{i,j}(t_{0})\right)}_{N\times N}$$\{L,R^{*}\}=ArgMin\left({\left\|S_{k}(t_{0})\cdot (LR^{*})-D_{k}(t_{0})\right\|}_{F}^{2}+\lambda \left({\left\|L\right\|}_{F}^{2}+{\left\|R^{*}\right\|}_{F}^{2}\right)+{\left\|H(t_{0})LR^{*}\right\|}_{F}^{2}+{\left\|LR^{*}T\right\|}_{F}^{2}\right)$${\hat{D}}_{k}(t_{0})=LR^{*}$**end for****return**${\hat{D}}_{1}(t_{0}),\cdots ,{\hat{D}}_{K}(t_{0})$

The adjacency matrix at $t_{0}$ is first defined as follows:

(17) $$H^{\prime }(t_{0})={\left(h_{i,j}^{\prime }(t_{0})\right)}_{N\times N}=\left\{ \begin{array}{l} 1{\;\mathrm{if}\;n}_{i}{\;\mathrm{and}\;n}_{j}\;\mathrm{are}\;\mathrm{neighbors}\;\mathrm{at}\;t_{0}\\ 0\;\mathrm{otherwise} \end{array}\right.,$$

where $n_{i}$ and $n_{j}$ are neighbors if their distance is less than threshold d. Then,

(18) $$H(t_{0})={\left(h_{i,j}(t_{0})\right)}_{N\times N}=\left\{ \begin{array}{ll} 0\; & \mathrm{if}\;\sum_{j=1}^{N}h_{i,j}^{\prime }(t_{0})=0\\ 1\; & \mathrm{else}\;\mathrm{if}\;i=j\\ -\frac{h_{i,j}^{\prime }(t_{0})}{\sum_{j=1}^{N}h_{i,j}^{\prime }(t_{0})} & \;\mathrm{otherwise} \end{array}\right..$$

According to [38], we set $T=Toeplitz{\left(0,1,-2,1\right)}_{L\times L}$, i.e.,

(19) $$T={\left[\begin{array}{cccc} 1 & -2 & 1 & 0\\ 0 & 1 & -2 & 1\\ 0 & 0 & 1 & -2\\ \vdots & \ddots & \ddots & \ddots \end{array}\begin{array}{c} \cdots \\ \vdots \\ \vdots \\ \ddots \end{array}\right]}_{L\times L}.$$

The sensory data reconstruction process is shown in Algorithm 3. In this algorithm, $ArgMin(f(L,R^{*}))$ finds the L and $R^{*}$ that minimize $f(L,R^{*})$.

## 5. Performance Evaluation

The proposed SR algorithm was tested on a real-weather data set to analyze its performance. The data set used in the experiment is the hourly observation data of Chinese ground meteorological stations acquired from the National Meteorological Information Center. We used temperature data of 145 meteorological stations in Sichuan Province from 01:00 on March 27, 2019, to 12:00 on March 27, 2019. We emulated collecting these data. Firstly, we tested the overall performance of the SR algorithm and analyzed the effect of clustering, sampling, and reconstruction processes on data collection performance. Secondly, we compared the SR algorithm with two existing representative approximate data collection algorithms in terrestrial IoT/WSN.

In the sampling stage, we set the time dimension curvature and the spatial dimension curvature to have the same weight in the composite curvature (i.e., $w_{1}=w_{2}=1$).

### 5.1. SR Algorithm Performance Analysis

The SR algorithm needs to maximize data collection precision under a certain data collection ratio. Therefore, we first test the overall performance of the SR algorithm. Then, in order to analyze the effect of clustering, sampling, and reconstruction processes on the performance, we analyze the data collection precisions under a different number of clusters, sampling methods, and reconstruction methods.

#### 5.1.1. Overall Performance

As shown in Table 3, we test the data collection precisions under different data collection ratios. Here, we set the number of clusters to 3. Overall, the SR algorithm can achieve high data collection precision under the tested data collection ratios. For example, when r=40%, the data collection precision is 0.8220; when r=90%, the data collection precision reaches 0.9852. The higher the data collection ratio, the higher the data collection precision. This is because when the data collection ratio is high, more data can be directly obtained, and the reconstruction precision of uncollected data is also higher.

In our experiments, the location selection of the meteorological stations has been carefully considered, which limits the data collection precision at lower data collection ratios. In many scenarios, the spatial density of the network deployment is superfluous, and higher data collection precision can be achieved with a certain data collection ratio.

#### 5.1.2. Effect of the Number of Clusters

**Figure 5:**
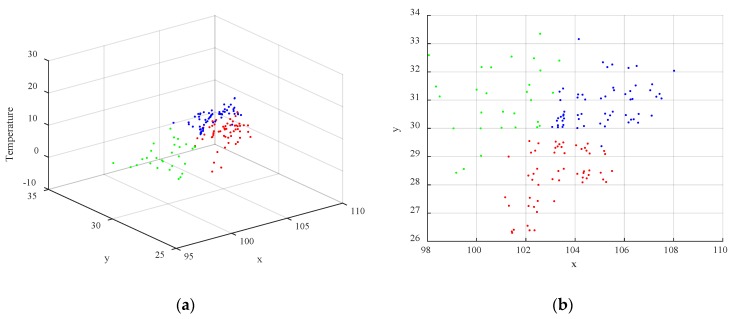
Clustering result (K=3): (**a**) 3D view of the clustering result; (**b**) 2D view of the x–y plane of the clustering result.

For the SR algorithm, the performance will be affected by the number of clusters K. When K=3, the corresponding result of clustering when data collection is performed at 01:00 on March 27, 2019, is shown in Figure 5. In the figure, the different colors of the points indicate that the points belong to different clusters. Figure 5a shows the 3D view, and Figure 5b shows the corresponding 2D view the of x–y plane.

**Figure 6:**
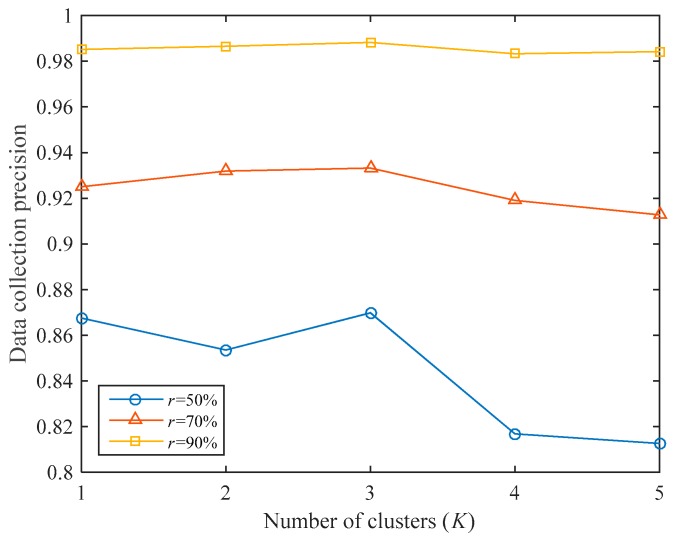
The effect of the number of clusters on the data collection precision.

As shown in Figure 6, the impact of the number of clusters under different data collection ratios is analyzed. If K=1, clustering will not be performed. It can be seen that the number of clusters will greatly impact data collection precision under the same data collection ratio. If r is small (for example, r=50%), there are few sampled nodes, and the data collection precision is significantly affected by the number of clusters; if r is large (for example, r=90%), since there are many sampled nodes, data collection precision is hardly affected by the number of clusters.

If K=3, the data collection precision thresholds are the highest under different r, which are 0.8698, 0.9332, and 0.9882, respectively. If K=5, the data collection precisions are 0.8126, 0.9127, and 0.9842, which are even worse than when clustering is not performed (the data collection precisions are 0.8675, 0.9250, and 0.9851, respectively). Therefore, according to the different collection tasks, we need to set the number of clusters reasonably. In this experiment, we think that setting the number of clusters to 3 is the most appropriate scenario.

#### 5.1.3. Effect of Sampling Methods

**Figure 7:**
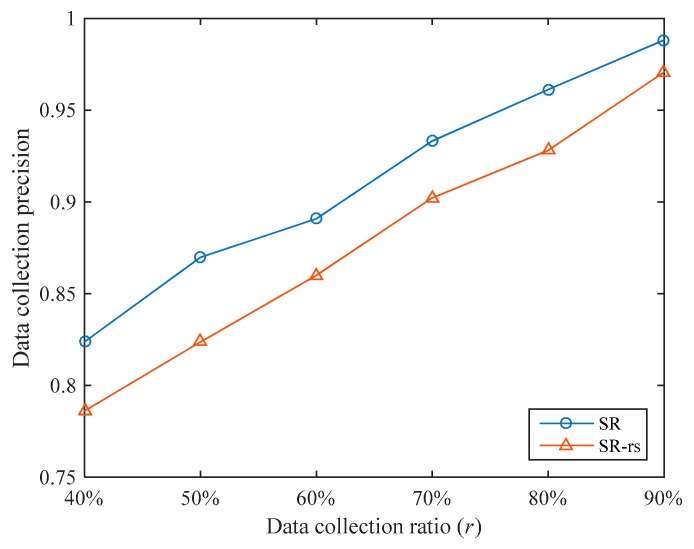
Comparison of data collection precisions between SR and the SR algorithm with random sampling (SR-rs).

In the sampling stage, we optimized the sampling method. In order to evaluate the effectiveness of this optimization, we compare the sampling method used in SR with random sampling, as shown in Figure 7. The comparison algorithm uses random sampling during the sampling stage, and the other parts are exactly the same as the SR algorithm, which is denoted as SR-rs. The number of clusters for both algorithms is set to 3.

Under different data collection ratios, the SR algorithm’s data collection precision is 3.80% higher than that of the SR-rs algorithm on average. Therefore, the optimization in the sampling stage is effective. In addition, overall, the improvement of SR’s data collection precision compared with SR-rs is gradually reduced as r increases. For example, when r=40%, SR’s data collection precision is 4.80% higher than that of SR-rs, and when r=90%, the improvement is only 1.80%. This is because as r increases, the number of sampled nodes gradually increases, and the difference between the sampled nodes of SR and SR-rs gradually decreases.

#### 5.1.4. Effect of Reconstruction Methods

In the reconstruction stage, we use the ST-CS technology to recover the data of the unsampled nodes through the sampled data. We can also use other methods to recover data; a typical method is two-dimensional interpolation. We compared the ST-CS with two-dimensional interpolation. The comparison algorithm uses the “v4” method in Matlab for interpolation calculation in the reconstruction stage and the other parts are exactly the same as the SR algorithm, which is denoted as SR-2di. We also set the number of clusters to 3 for the two algorithms.

**Figure 8:**
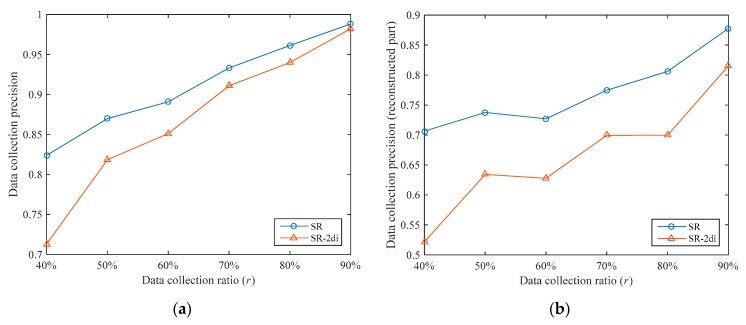
Comparison of data collection precisions between SR and the SR algorithm with two-dimensional interpolation in the reconstruction stage (SR-2di): (**a**) overall data collection precisions of SR and SR-2di; (**b**) data collection precisions of the reconstructed part in SR and SR-2di.

The data collection precisions of the two algorithms under different data collection ratios are shown in Figure 8a. It can be seen that the data collection precision thresholds of the SR algorithm are higher under different data collection ratios. The data collection precision of the SR algorithm is 5.30% higher than that of the SR-2di algorithm on average. In order to analyze the data reconstruction performances of the two algorithms, we separately compared the data collection precisions of the reconstructed part (i.e., the unsampled nodes), as shown in Figure 8b. Under different data collection ratios, the data collection precision of the reconstruction part in SR is 16.58% higher than that of SR-2di on average. This shows that ST-CS has better data reconstruction performance than two-dimensional interpolation. This is because ST-CS simultaneously exploits the correlations of sensory data in temporal and spatial dimensions, while two-dimensional interpolation only utilizes the correlations in spatial dimensions.

In addition, whether it is the overall data collection precision or the data collection precision of the reconstructed part, the increase of SR compared with SR-2di decreases with the increase of r. This is because as r increases, the number of sampled nodes gradually increases. On the one hand, the number of unsampled nodes is gradually reduced, and the impact of their data collection precisions on the overall data collection precision is gradually reduced. On the other hand, when the number of sampled nodes increases, the difference in data reconstruction performances between the two reconstruction methods is gradually reduced.

### 5.2. Comparisons with Other Algorithms

In this section, we compare the SR algorithm with two existing approximate data collection algorithms. Since the query-driven algorithms are not general purpose data collection algorithms, we do not compare with them. We selected a representative algorithm for model-based algorithms and compressed-sensing-based algorithms, respectively, namely the Ken algorithm proposed in [10] and the CDG algorithm proposed in [17]. We have already introduced the two algorithms in Section 2. The specific settings of the two algorithms in the experiment are as follows.

Ken: The main purpose of this algorithm is to reduce the communication overhead between sensor nodes and the sink. In the experiment, we use 145 meteorological stations as sensor nodes and the data center as the sink node, thus reducing the data that the space-based information network needs to transmit. The models running in meteorological stations and data centers are time-varying multivariate Gaussian models. The 145 meteorological stations are divided into 49 disjointed cliques. Each clique selects a root node, which runs a local model, and the remaining nodes in the clique send data to the root node.

CDG: We use 145 meteorological stations as sensor nodes and select a meteorological station as the sink. We assume that there are links between meteorological stations. The sensory data is first transmitted to the sink after compression, and the sink transmits the compressed data to the data center through the space-based information network. The sparse domain of the sensory data is obtained through an overcomplete dictionary training. The training set is temperature data of 145 meteorological stations from 00:00 on March 21, 2019, to 00:00 on March 27, 2019.

In the contrast experiment, the number of clusters in the SR algorithm is set to 3. We compare the data collection precision, data collection ratio, and communication overhead in the internode network. For the convenience of comparison, in the Ken algorithm, we define the data collection ratio as the proportion of sensory data that need to be sent to the sink because the sensory values and the values predicted by local models are not within the error range; in the CDG algorithm, we define the data collection ratio as the ratio of the number of compressive sensing measurements to the number of sensory data. In the internode network, one communication overhead is generated when sensory data is transmitted over one hop.

#### 5.2.1. Data Collection Precision

**Figure 9:**
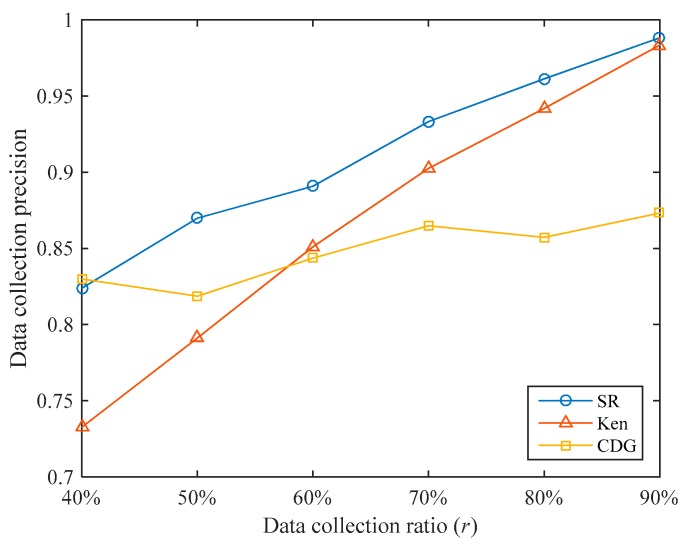
Comparison of data collection precisions of the three algorithms.

The data collection precisions of the three algorithms under different data collection ratios are shown in Figure 9. Overall, SR’s data collection precisions are higher than that of Ken and CDG. Under the data collection ratios tested, the data collection precision of SR is 5.33% and 7.21% higher than that of Ken and CDG, respectively. It can be seen that the SR algorithm can achieve higher data collection precision under the same data collection ratio.

When the data collection ratio is small, Ken’s data collection precision is low. For example, when r=40%, Ken’s data collection precision is only 0.7326, which is lower than the 0.8238 of SR. This is because when the data collection ratio is small, the error range of the predicted value is set to be larger, few sensory data are transmitted to the sink, and the untransmitted sensory values have larger errors. When the data collection ratio increases, Ken’s data collection precision increases rapidly and gradually approaches SR. For example, when r=90%, Ken’s data collection precision reaches 0.9833. This is because when the data collection ratio is large, the error range of the predicted value is set to be smaller, more sensory data are transmitted to the sink, and the errors of the untransmitted sensory values are smaller.

When the data collection ratio is small, the CDG algorithm can achieve high data collection precision. For example, when r=40%, the data collection precision of CDG reaches 0.8299. This is because we have specially trained for different data collection ratios and the training data set is close to the test data set in time, so higher data collection precision can be obtained at a lower data collection ratio. However, as the data collection ratio increases, the increase of CDG’s data collection precision is not obvious. For example, when r=90%, the data collection precision of CDG is only 0.8732. This shows that the sparsity of the sensory data matrix is not obvious in our data set.

#### 5.2.2. Data Collection Ratio

**Figure 10:**
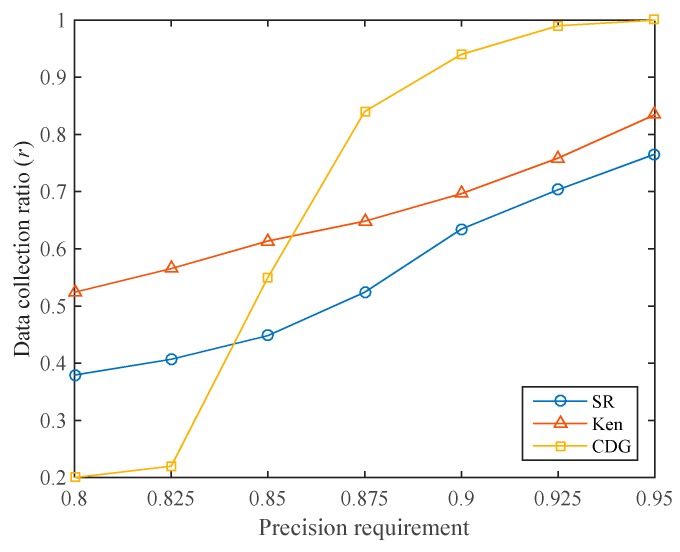
Comparison of data collection ratios of the three algorithms.

In many applications, the user will specify the data collection precision requirement, and we hope to reach the precision requirement with a lower data precision ratio. For different data collection precision requirements, the data collection ratios of the three algorithms are shown in Figure 10. It can be seen that the CDG algorithm has the lowest data collection ratio when the required data collection precision is low (below 0.85). For example, when the required data collection precision is 0.8, the data collection ratios of SR, Ken, and CDG are 37.93%, 52.41%, and 20.00%, respectively. As mentioned earlier, this is because CDG can achieve high data collection precision at a low data collection ratio. However, when the precision requirement increases, the data collection ratio required by the CDG algorithm increases dramatically, much higher than that of SR and Ken. This is consistent with Figure 9. Since the sparsity of the sensory data matrix is not high, the CDG algorithm requires more compressive sensing measurements to meet the required data collection precision.

The data collection ratio of the SR algorithm is kept at a low level. Under different data collection precision requirements, the SR algorithm requires lower data collection ratios than the Ken algorithm.

#### 5.2.3. Communication Overhead in Internode Network

**Figure 11:**
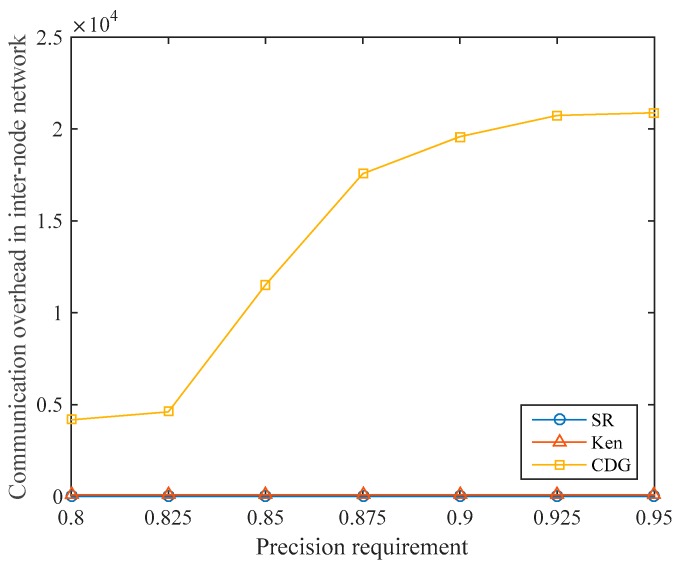
Comparison of communication overhead in the internode networks of the three algorithms.

Under different data collection precision requirements, the communication overhead in the internode network of the three algorithms is shown in Figure 11. For the Ken algorithm, in each data collection cycle, the non-root nodes in each clique send sensory data to the root node, which brings communication overhead to the internode network. After the clique partitioning, the root nodes, and the routing paths are determined, the communication overhead in the internode network is fixed in every cycle. In our experiments, the communication overhead in the internode network of Ken is always 96.

We assume that the number of compressive sensing measurements is M and the number of nodes is N in the CDG algorithm. Since the amount of data that each node needs to transmit is M and N−1 nodes need to transmit data to the sink node, the communication overhead in the internode network is M(N−1). Because N=145, the communication overhead in the terrestrial network of CDG is only related to the number of compressive sensing measurements M. When the precision requirement increases, the communication overhead in the internode network increases sharply due to the rapid increase in the data collection ratio required by CDG.

In the SR algorithm, since the sampled nodes directly transmit data to the satellite, communication between the ground nodes is not required, so the communication overhead in the internode network is always zero. As mentioned earlier, the nodes in S-IoT are usually distributed over a wide area, and there may not be any stable links between the nodes, or even no links at all. Therefore, the SR algorithm has better adaptability in S-IoT.

In the SR algorithm, the sampled nodes directly report the sensory data to satellites without relying on the internode network. Moreover, the selected node only needs to report data, and there is no additional operation. No action is required on the unselected nodes. Node clustering, sampling node selection, and sensory data reconstruction are all performed in data centers, which are rich in resources. Therefore, the SR algorithm has better data collection performance and is more suitable for S-IoT.

## 6. Conclusions and Future Work

In S-IoT, a single satellite usually covers a vast area and needs to provide data transmission services for a large number of nodes. Even worse, the bandwidth of the satellite–ground link is usually low, and the uplink and downlink bandwidth is extremely asymmetric. This makes it difficult for S-IoT to achieve exact data collection and poses a great challenge for efficient data collection in S-IoT. In this paper, we propose the SR algorithm, an approximate data collection algorithm for S-IoT. Due to the limited uplink bandwidth, the SR algorithm only samples the sensory data of some nodes and then reconstructs the unacquired sensory data. In order to obtain higher data collection precision under a certain data collection ratio, the SR algorithm is optimized from two aspects: (1) in the sampling phase, the sampling node selection is optimized by using the curvature characteristics of the sensory data in time and space dimensions; (2) in the reconstruction phase, we innovatively use ST-CS technology to fully utilize the spatiotemporal correlation of sensory data to reconstruct sensory data. We use real-weather data set to test the proposed SR algorithm. The experiments verify the effectiveness of the sampling node selection optimization and ST-CS-based sensory data reconstruction in the SR algorithm. Moreover, compared with the existing approximate data collection algorithms in the terrestrial IoT/WSN, the SR algorithm is more suitable for S-IoT, and can achieve efficient data collection under the condition of extremely limited uplink bandwidth.

In the follow-up work, we will further study the efficient data collection in S-IoT. For the sampling phase of the SR algorithm, we plan to study sampling methods such as uniform sampling and density sampling in order to further optimize the selection of sampling nodes. For the reconstruction phase of the SR algorithm, we will try to optimize the spatial constraints and time constraints in ST-CS according to the characteristics of the spatiotemporal correlation of sensory data in S-IoT to obtain higher data reconstruction precision. Furthermore, we will explore other data reconstruction methods. In addition, for multimedia data, we will consider using artificial intelligence methods such as neural networks and machine learning to preprocess the data at access gateways, ground stations, satellites, and other points. For example, we could perform key target recognition and retain only the data containing the target of interest, or directly extract the parameter values of the target and convert the multimedia data into numerical data.

## Figures and Tables

**Table 1:** Comparison of space-based Internet of Things (S-IoT) and terrestrial IoT/WSN.

Network Type	Coverage of a Base Station	Number of Nodes Connected to a Base Station	Internode Link	Common Connection Mode between Nodes and Base Stations
S-IoT	Thousands of kilometers	Tens of thousands	No stable link, or even no links	Direct connection
Terrestrial IoT	Tens of kilometers	Hundreds	Exists	Multi-hop connection via internode links
WSN	Hundreds of meters	Tens	Exists	Multi-hop connection via internode links

**Table 2:** The reconstructed sensory data of $n_{i}$ at $t_{0}-T$ and the previous $T_{C}$.

t	$t_{0}-(L-1)T$	$t_{0}-(L-2)T$	…	$t_{0}-T$
$d_{i}^{\prime }(t)$	$d_{i}^{\prime }(t_{0}-(L-1)T)$	$d_{i}^{\prime }(t_{0}-(L-2)T)$	…	$d_{i}^{\prime }(t_{0}-T)$

**Table 3:** Data collection precision under different data collection ratios.

r	40%	50%	60%	70%	80%	90%
A	0.8220	0.8658	0.8908	0.9332	0.9612	0.9852
